# 

**Published:** 1906-02-15

**Authors:** 


### CONTENTS.

Event and Comment - - - - - - - - - - 55

~~Professional Ethics,~~ - - - By T. R. Buttrick, D.D.S. 59

Pressure Anesthesia, - - - - - - By Geo. Zederbaum 80

Making an Aluminum Plate, - - - - By Geo. D. Sitherwood 93

Bibliography - - - - - - - - - - 95

Obituary, - - - - - - - - - - - - 97

Indents - - - - - - - - - - - - 99

Announcements - - - - - - - - - - - 107

